# Fluid accumulation, recognition and staging of acute kidney injury in critically-ill patients

**DOI:** 10.1186/cc9004

**Published:** 2010-05-06

**Authors:** Etienne Macedo, Josée Bouchard, Sharon H Soroko, Glenn M Chertow, Jonathan Himmelfarb, T Alp Ikizler, Emil P Paganini, Ravindra L Mehta

**Affiliations:** 1 Division of Nephrology and Hypertension, Department of Medicine, University of California San Diego San Diego, 200 West Arbor Drive, MC 8342, San Diego, CA 92103, USA; 2 Division of Nephrology, Department of Medicine, Stanford University School of Medicine, 780 Welch Road, Suite 106, Palo Alto, CA 94034, USA; 3 Kidney Research Institute, Division of Nephrology, Department of Medicine, University of Washington, 908 Jefferson St, Seattle, WA 98104, USA; 4 Division of Nephrology, Department of Medicine Vanderbilt University School of Medicine,638 Robinson Research Building, 2200 Pierce Avenue, Nashville, TN 37232-0146, USA; 5 Division of Nephrology, Department of Medicine, Cleveland Clinic Foundation, 9500 Euclid Avenue, Cleveland, OH 44195, USA

## Abstract

**Introduction:**

Serum creatinine concentration (sCr) is the marker used for diagnosing and staging acute kidney injury (AKI) in the RIFLE and AKIN classification systems, but is influenced by several factors including its volume of distribution. We evaluated the effect of fluid accumulation on sCr to estimate severity of AKI.

**Methods:**

In 253 patients recruited from a prospective observational study of critically-ill patients with AKI, we calculated cumulative fluid balance and computed a fluid-adjusted sCr concentration reflecting the effect of volume of distribution during the development phase of AKI. The time to reach a relative 50% increase from the reference sCr using the crude and adjusted sCr was compared. We defined late recognition to estimate severity of AKI when this time interval to reach 50% relative increase between the crude and adjusted sCr exceeded 24 hours.

**Results:**

The median cumulative fluid balance increased from 2.7 liters on day 2 to 6.5 liters on day 7. The difference between adjusted and crude sCr was significantly higher at each time point and progressively increased from a median difference of 0.09 mg/dL to 0.65 mg/dL after six days. Sixty-four (25%) patients met criteria for a late recognition to estimate severity progression of AKI. This group of patients had a lower urine output and a higher daily and cumulative fluid balance during the development phase of AKI. They were more likely to need dialysis but showed no difference in mortality compared to patients who did not meet the criteria for late recognition of severity progression.

**Conclusions:**

In critically-ill patients, the dilution of sCr by fluid accumulation may lead to underestimation of the severity of AKI and increases the time required to identify a 50% relative increase in sCr. A simple formula to correct sCr for fluid balance can improve staging of AKI and provide a better parameter for earlier recognition of severity progression.

## Introduction

The mortality rate in patients with severe acute kidney injury (AKI) ranges from 40% to 80%, despite advances in the management of ICU patients and improvement in dialysis techniques [1-5]. Minimal increases in serum creatinine (sCr) concentration are now recognized as clinically significant events and the severity of AKI has been associated with a progressive increase in mortality [6-8]. Current diagnostic and staging criteria for AKI are based on changes in sCr and require sequential measurements [9,10]. Given the exponential relation of sCr and glomerular filtration rate (GFR), significant decreases in GFR are reflected as small increases in sCr in the early phases of injury [11,12]. Consequently, factors influencing sCr could affect time to recognition of AKI and lead to underestimating the severity of renal dysfunction over the course of AKI. Aside from the well-recognized biological influences of age, muscle mass, catabolic rate and race [13,14], alterations in the volume of distribution of creatinine (V_Cr_) can in turn alter the sCr concentration.

Animal and human studies have suggested that the V_Cr _is roughly equivalent to total body water (TBW) [15,16]. Among critically-ill patients, especially following surgery or resuscitation for sepsis or other conditions requiring massive volume expansion (*e.g.*, burns, pancreatitis, cancer chemotherapy or bone marrow transplantation), the increase in TBW can reach more than 10% within 72 hours [17,18]. Thus, in addition to its dependence on creatinine generation and clearance (reflecting muscle mass breakdown and kidney function, respectively), the accuracy of sCr measurements as a reflection of kidney function also depends on TBW. All else equal, higher TBW results in lower sCr, which can lead to underestimation of severity of kidney injury.

The Program to Improve Care in Renal Disease (PICARD) was a multi-center cohort study examining patient characteristics and practice patterns associated with adverse and favorable outcomes in patients with AKI [19]. Laboratory studies and fluid status were obtained daily throughout the ICU stay. Using data from PICARD, we hypothesized that a positive cumulative fluid balance would underestimate the severity of AKI and increase the time to appropriately stage the disease.

## Materials and methods

### Study participants

From February 1999 to August 2001, the PICARD study personnel evaluated for potential study participation all patients from five academic medical centers who underwent a nephrology consultation for AKI in the ICU. The study protocol was approved by the institutional review boards of the participating institutions and informed consent was obtained from all patients or their legal representatives. AKI was defined as an increase in sCr of 0.5 mg/dL or more for baseline sCr of less than 1.5 mg/dL or an increase in sCr of 1.0 mg/dL or more for baseline sCr of 1.5 mg/dL or more and less than 5.0 mg/dL. Chronic kidney disease (CKD) status was determined at enrollment for each patient by evaluating available clinical and laboratory data and history. At time of enrollment, patients were identified as having CKD if they had evidence of elevated sCr, proteinuria, or an abnormal renal ultrasound within a year prior to the index hospitalization. Patients were classified as 'CKD with AKI' if they met criteria for CKD as defined above. All remaining patients were considered as 'new-onset AKI'. A complete description of generation of the PICARD cohort, data elements, data collection, and management strategies have been previously described [19]. Of the 618 patients included in the database, 398 required dialysis, some as early as at the first day of consultation. We identified 253 AKI patients with three to seven days of consecutive increase, with no fluctuations in sCr before dialysis initiation. We excluded patients with one day of missing data for sCr during that phase. sCr was measured at least once every 24 hours. In this analysis, we compared the first sCr value available each day with the first sCr value in the observational period (reference value).

### Weight and fluid balance

Admission weights were available in all patients and were utilized to estimate TBW. Daily fluid balance was determined from all intakes and outputs recorded. No correction was made for insensible losses. Cumulative fluid balance was computed by summing the daily fluid balances. In the subset of patients with available daily weights, the change in daily weight was compared with daily fluid balance.

### Correction of sCr for fluid balance

sCr values were adjusted according to the cumulative daily fluid balance using the formula [20]:

adjusted creatinine = sCr x correction factor

Correction factor = (hospital admission weight (kg) x 0.6 + Σ (daily cumulative fluid balance (L))) / hospital admission weight x 0.6.  

### Calculation for underestimation

**Figure 1:**
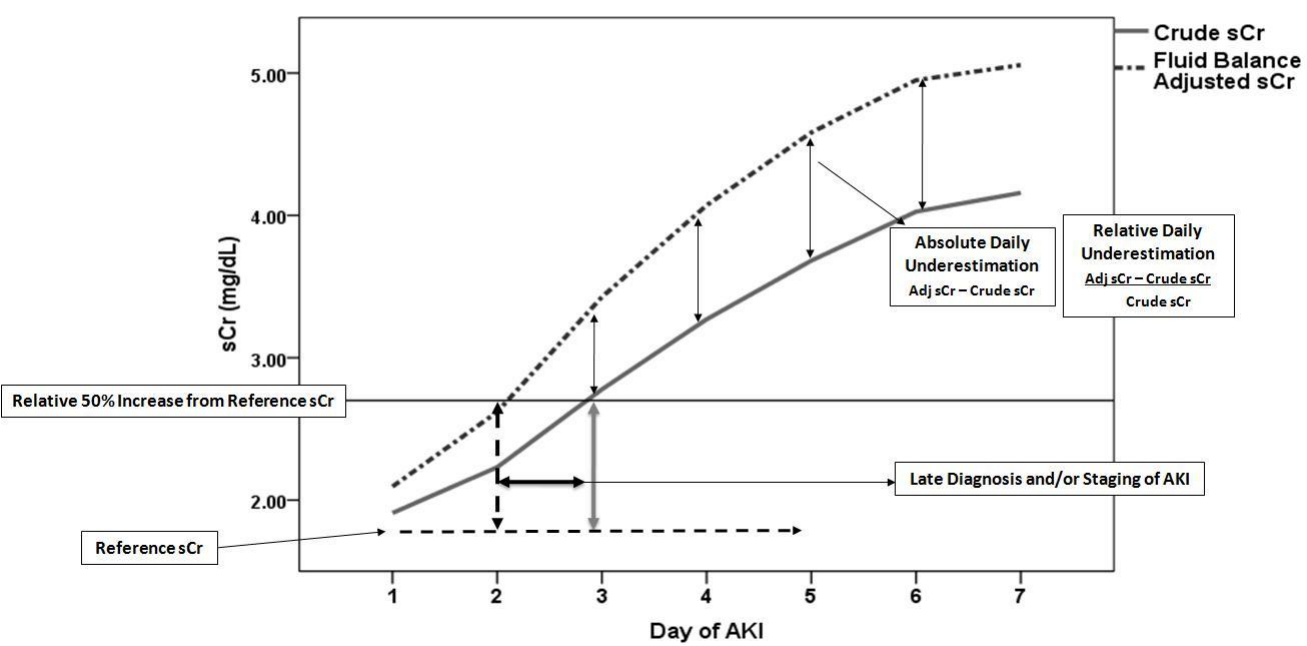
**Difference between mean crude and adjusted serum creatinine during the follow-up period (late recognition of severity group)**. For conversion of creatinine expressed in conventional units to standard units, multiply by 88.4. AKI: acute kidney injury; sCr: serum creatinine.

Underestimation was evaluated in two ways. First, we computed the differences between the daily adjusted and crude (measured) sCr values and expressed these as an absolute change in mg/dL and as a percentage of the crude value for the day (daily underestimation). Additionally, the time difference to reach a 50% relative increase from reference based on crude and adjusted sCr was calculated (Figure 1).

daily underestimation = adjusted sCr - crude sCr for the day   

% daily underestimation = (adjusted sCr - crude sCr for the day) / crude sCr for the day x 100  

Difference in time to recognize a 50% increase from reference sCr = day reached a relative 50% increase in sCr based on adjusted sCr - day reached a relative 50% increase in sCr based on crude sCr.

We considered a late recognition in severity progression when the interval to reach the 50% relative increase by the crude sCr and adjusted sCr was longer or equal to one day.

### Statistical analyses

Continuous variables were expressed as mean ± standard deviation or median and interquartile range (IQR), and compared using either the student's *t *test or Wilcoxon rank-sum test, as appropriate. Categorical variables were expressed as proportions and compared with the chi-squared. All statistical tests were two-sided and *P *< 0.05 was considered significant. Statistical analyses were conducted using SPSS 17.0 (Chicago, IL, USA).

## Results

Of 253 patients in the development phase of AKI with a consecutive increase in sCr, the mean age was 60 (± 16.2) years, 64% were male, and 15% were non-white. Thirty-one percent had a history of CKD. Mean body weight at hospital admission was 81.8 (± 20.3) kg. Median daily urine volume was 1295 mL (IQR 621 to 2145 mL) and 41% of the patients had an episode of oliguria (urine output less than 400 mL/24 hours) for at least one day. Changes in daily weight and daily fluid balance could be compared in 82 patients over 212 days and the correlation (r = 0.452; *P *< 0.001).

### Effect of fluid accumulation on serum creatinine

The median sCr on day 1 was 1.6 mg/dL (IQR 1.2 to 2.2) and increased to 3.9 mg/dL (IQR 2.8 to 5.6) at day 7. Over the study period, median cumulative fluid balance increased from 2.7 L (IQR 0.5 to 6.2) on day 2 to 6.5 L (IQR 1.1 to 11.3) on day 7 (Table 1). sCr concentrations adjusted for fluid balance were significantly higher at each time point and the difference from median crude and adjusted values progressively increased from 0.09 mg/dL to 0.65 mg/dL. This daily difference in sCr would translate to a median underestimation of 7.0% (IQR 1.3 to 16%), ranging from 2.1% (IQR 0 to 6.5%) after one day to 14.3% (IQR 4.6% to 27.9%) on day 6 (Table 1).

**Table 1:** Median daily cumulative fluid balance and serum creatinine (crude and fluid adjusted) in all patients

Median (IQR)	Day 1	Day 2	Day 3	Day 4	Day 5	Day 6	Day 7
**Cumulative FB (L)**	1.0 (-0.1-3.2)	2.7 (0.5-6.2)	3.7 (1.1-8.6)	4.9 (1.7-10.3)	5.6 (2.5-12.0)	6 (1.9-13.1)	6.5 (1.1-11.3)
**Crude sCr** **(mg/dL)**	1.60 (1.2-2.2)	2.10 (1.5-2.8)	2.80 (2.1-3.7)	3.30 (2.6-4.6)	3.80 (2.9-5.5)	3.90 (2.9-5.5)	3.90 (2.8-5.6)
**FB adjusted sCr** **(mg/dL)**	1.69 (1.2-2.3)	2.24 (1.6-3.1)	2.99 (2.3-4.2)	3.79 (2.8-5.2)	4.29 (3.2-6.3)	4.44 (3.4-6.3)	4.55 (3.4-6.6)
**% Underestimation**	2.1 (0-6.5)	5.4 (0.9-13.1)	8.3 (2.18-17.9)	10.5 (3.2-21.8)	13.4 (4.8-25.8)	14.3 (4.6-27.9)	13.6 (2.8-26.9)

FB: fluid balance; IQR: interquartile range; sCr: serum creatinine.

For conversion of creatinine expressed in conventional units to standard units, multiply by 88.4.

*% Daily underestimation = (Adjusted sCr - crude sCr for the day) crude sCr for the day *× 100

### Patients' characteristics and outcomes among those with and without late recognition of severity progression

**Figure 2:**
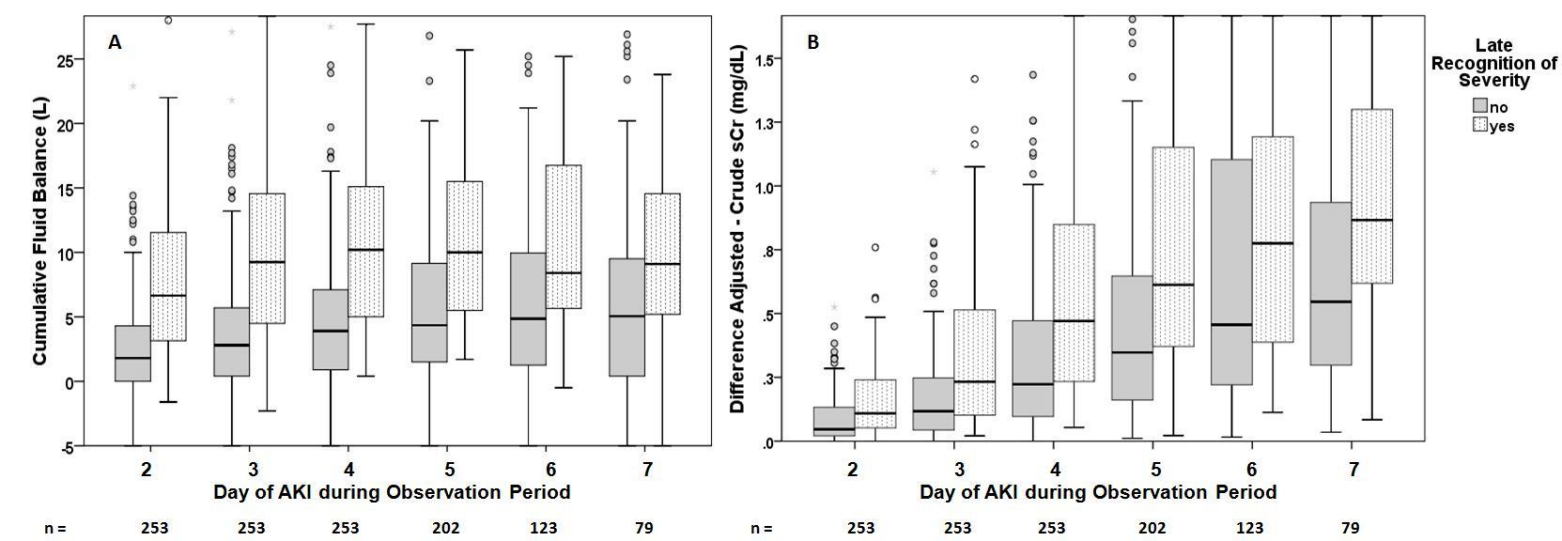
**(a) Cumulative fluid balance and (b) difference between adjusted and crude sCr during the observation period in patients with and without late recognition of severity**. (a) * *P *< 0.001; ** *P *= 0.003; *** *P *= 0.007. **(b) ***P *< 0.001 all days. AKI: acute kidney injury; sCr: serum creatinine.

In addition, 64 (25%) patients had an interval of one day or more to reach a relative 50% increment from reference creatinine comparing crude and fluid adjusted sCr (Table 2). In 24 (9%) patients this interval was two or more days. These 64 patients (late recognition) had a higher cumulative fluid balance and consequently a greater difference between crude and adjusted sCr starting on day 1 (Figures 2a and 2b). Dialysis was initiated more frequently in patients with late recognition (71% vs. 58% in patients with no late recognition, *P *= 0.061). In-hospital mortality was not significantly different between the two groups (40% with late recognition vs. 35% without late recognition, *P *= 0.45) (Table 2).

**Table 2:** Patients' characteristics and outcomes -- All patients and patients with and without late recognition of severity

	All patients	Patients with late recognition	Patients without late recognition
	
	n = 253	n = 64	n = 189
**Race - Caucasian**	85%	87%	84%
**Race - African American**	6.3%	3.1%	7.4%
**Gender - male**	63%	59%	65%
**Serum creatinine - day 1- mg/dL -- median (IQR)**	1.6 (1.2-2.2)	1.65 (1-2.4)	1.60 (1.2-2.2)
**CKD status -- n (%)**	79 (31)	16/62 (25)	63/187 (33)
**Daily urine volume - mL -- median (IQR)**	1295 (621-2145)	1151 (475-1955)	1338 (670-2200)*
**Oliguria <400 ml/24 hours - n (%)**	103 (40.7)	31/64 (48)	72/189 (38)
**Daily fluid balance (L/24 h) - median (IQR)**	0.7 (-0.3-2.3)	1.6 (0.0-3.6)	0.5 (-0.4-2.0)**
**Total fluid accumulation (L) - median (IQR)**	4.9 (0.6-10.9)	11.2 (5.5-16.5)	3.2 (-0.7-7.3) **
**Total fluid accumulation as % of body weight - median (IQR)**	6.4 (0.7-14.4)	15.6 (7.0-21.3)	4.2 (-.8-10.3) **
**Need for dialysis**	157/253 (52)	46/64 (71)	111 (58) ***
**Hospital mortality**	93/253 (36)	26/64 (40)	67/189 (35)

* *P *= 0.003; ** *P *< 0.001; *** *P *= 0.061 (not significant)

CKD: chronic kidney disease; IQR: interquartile range.

For conversion of creatinine expressed in conventional units to standard units, multiply by 88.4.

## Discussion

Fluid administration is a common and required component of the management of critically-ill patients and has recently focused on goal-directed resuscitation with early volume expansion in the ICU course. These strategies frequently result in a relative increase in body weight of 10 to 15% or more, sometimes doubling the TBW in a short period of time [18,21]. Moran and Myers previously demonstrated the effect of fluid accumulation on sCr concentrations and showed that increasing the TBW alters the volume of distribution of sCr, resulting in potential for overestimation of the level of kidney function [20]. As the assessment of AKI is largely based on changes in sCr, we extended the observations of Moran and Myers using a cohort of critically-ill patients with AKI. We hypothesized that fluid accumulation would underestimate the severity of renal dysfunction based on sCr and increase the time to detect a change in severity of injury.

Previous studies have shown varying incidences of AKI depending on the diagnostic method used, but none has compared the assessment of severity of AKI in relation to cumulative fluid balance [22,23]. In this cohort fluid accumulation progressively increased in patients as kidney function declined. The progressive increase in fluid accumulation resulted in differences as large as 1 mg/dL between sCr concentrations corrected for cumulative fluid balance and crude sCr.

Early recognition of AKI has become an area of intensive investigation after studies showing that even small increases in sCr are associated with increases in mortality and morbidity. A more precise determination of AKI severity is an important goal, because mortality with and complications of AKI appear to be proportional to its severity [8,24]. For example, Chertow and colleagues showed a 6.5-fold increase in the odds of death for patients with a 0.5 mg/dL increase in sCr [8]. In pediatric patients with acute decompensated heart failure, Goldstein and colleagues found that a rise in sCr of 0.3 mg/dL or more was associated with a seven-fold increased risk of in-hospital death [25]. Additionally, several studies have now shown that the change in severity stage of AKI (acute kidney injury network (AKIN) or risk, injury, failure, loss of kidney function and end-stage renal failure (RIFLE)) is associated with an incremental risk for mortality [2,26,27]. An accurate assessment of AKI severity is essential to developing approaches for earlier intervention, to correct reversible factors, and mitigate the downstream effects of AKI. We tested this concept by establishing a criterion for significant underestimation as equivalent to the minimum criterion for AKIN stage 1 and RIFLE risk categories as these have been associated with adverse outcomes [2,26,27]. We found that following adjustment for fluid accumulation would have allowed one-quarter of patients to be recognized as having reached a percentage change in sCr one day earlier. The masking of AKI severity by volume expansion may be especially problematic in settings where the sCr is rising relatively slowly owing either to lower creatinine generation (e.g., as might be expected in the elderly or patients with less muscle bulk) or to more modest overall injury.

Our findings have potential practical implications. Patients included in this study were all analyzed during the phase of rising sCr. In this situation, clinical decisions for interventions (wait and see, consultation, diuretics, dialysis) are based on ascertaining the absolute level of sCr and the rate of change over a set period of time. Clinicians generally assess the daily change in sCr and change over the duration of the episode to gauge the severity of AKI at any time point. Perhaps individual values for sCr should be adjusted for the cumulative fluid balance to provide a more accurate assessment of the current severity of AKI on any given day. sCr level is a function of creatinine production and renal excretion, and the increment in sCr levels on each day is an approximation of the catabolic rate. In AKI critically-ill patients, the catabolic rate is likely to be increased and the creatinine production is unstable. Correcting sCr for fluid balance prior to calculating the creatinine production would provide more precise evaluation of the catabolic state to ascertain the true change in creatinine that could be masked by significant fluid accumulation.

As shown in Figure 1, the difference in crude and adjusted sCr increases over time and reflects the need to assess cumulative fluid balance rather than daily fluid balance alone as the latter may be negative, positive or even on any given day. Additionally, comparison of the fluid adjusted sCr to the reference creatinine at any given point might lead to an earlier delineation of a change AKI staging. As an adequate assessment of AKI severity can lead to an earlier implementation of preventive and therapeutic strategies, such as avoiding radiocontrast or discontinuing potential nephrotoxic drugs, adjusting medication dosages, and correcting hemodynamic status in an early phase of kidney injury, earlier recognition could be of value [2,28-30].

This study has several strengths. The PICARD cohort was assembled from five academic medical centers geographically distributed across the USA, with demographic and clinical characteristics reasonably representative of critically-ill patients with AKI. In contrast to many other studies where information was collected upon initial review or around the time of initiation of dialysis, data from patients enrolled in PICARD were collected from three days preceding the day of AKI diagnosis throughout their ICU course. Although several years have passed since the PICARD data were collected, many of the same issues facing patients with AKI remain. PICARD affords us with extraordinarily detailed clinical data on a relatively large cohort. Other administrative databases, although powerful, lack the clinical detail available in PICARD.

This study also has several important limitations. First, the problem of under-ascertainment may have led to even more underestimation or late recognition of AKI by the more stringent enrollment criteria employed in PICARD (requiring a 0.5 mg/dL increase in sCr in contrast to an 0.3 mg/dL increase in AKIN). Indeed, underestimation of AKI severity due to dilution of sCr by volume accumulation is likely to be more common in patients with mild AKI than in more severe cases, where the sCr rises rapidly and to a sufficient level (e.g., >2 mg/dL) where it is easily recognized in spite of dilution. However, even in these circumstances there is an incremental change in the time to detect a change in severity of AKI. Daily weight was available in a small proportion of patients and daily fluid balance is certainly subject to variation, because it does not account for insensible losses. However, daily fluid balance is a widely used method of assessing changes in volume status and it showed a positive correlation with weight increase in our cohort. We did not differentiate the type and nature of fluid given (colloid, crystalloids or nutritional supplements) and we could not ascertain all of the reasons for fluid accumulation. It would have been informative to know whether fluid (rather than pressors) was administered for the purpose of treating hypotension or in conjunction with other medications or nutritional support. We could not calculate creatinine production in our patients as we did not have urinary creatinine measurements in the majority of these patients. Finally, although volume accumulation clearly alters sCr in the 'development' phase of AKI [31] and could change practice patterns, it would also be informative to consider volume effects during the 'recovery' phase of AKI when sCr stabilizes and begins to decline. Although arguably less critical to patient outcomes, appropriately recognizing the pace of recovery by the decline in adjusted rather than crude sCr could help to rationalize inpatient and follow-up care after resolution of critical illness.

## Conclusions

In critically-ill patients, a positive fluid balance may lead to underestimation of the severity of AKI and delay the recognition of a 50% relative increase in sCr. The use of a simple formula to correct for fluid balance may allow for a more accurate determination of AKI severity in critically-ill patients. Future studies, including observational cohort studies and randomized clinical trials of patients with AKI, should consider the influence of fluid balance on sCr when designing inclusion criteria for participation.

## Key messages

• Positive fluid balance is common in the development phase of AKI

• Fluid accumulation increases the TBW and alters the volume of distribution of sCr

• The severity of AKI can be underestimated in patients with net positive fluid balance in the development phase of AKI

• Underestimation of sCr values can delay the recognition of a 50% relative increase in sCr

• Correcting sCr for fluid accumulation may allow for a more accurate determination of AKI severity in critically-ill patients

## Abbreviations

AKI: acute kidney injury; AKIN: Acute Kidney Injury Network; CKD: chronic kidney disease; GFR: glomerular filtration rate; IQR: interquartile range; PICARD: Program to Improve Care in Renal Disease; RIFLE: risk, injury, failure, loss of kidney function and end-stage renal failure; sCr: serum creatinine; TBW: total body water; V_Cr_: volume of distribution of creatinine.

## Authors' contributions

EM, JB, GMC, and RLM were involved in the conception, design, analysis and interpretation of data, drafting the article and revising it critically for important intellectual content and final approval of the version to be published. SS was involved in the analysis and interpretation of data, revising the article for important intellectual content and final approval of the version to be published. JH, TAI, and EPP were involved in the acquisition, analysis and interpretation of data, revising the article for important intellectual content and final approval of the version to be published.
